# Ineffective ventilation in a very low birth weight infant with H-type Tracheoesophageal Fistula with bilateral chemical pneumonitis. A lesson learned

**DOI:** 10.12669/pjms.40.2(ICON).8958

**Published:** 2024-01

**Authors:** Faraz Ahmad, Sana Niaz, Naveed Durrani, Vikram Kumar

**Affiliations:** 1 Dr. Faraz Ahmad Junior Consultant Neonatology, Sindh Institute of Child Health and Neonatology, Karachi, Pakistan; 2 Dr. Sana Niaz Consultant Neonatologist, Indus Hospital and Health Network, Karachi, Pakistan; 3 Dr. Naveed Durrani Neonatologist Sidra Medicine, Doha Qatar. Assistant Professor, Clinical Pediatrics, Weill Cornell Medicine, Doha, Qatar; 4 Dr. Vikram Kumar Consultant Neonatologist, Indus Hospital and Health Network, Karachi, Pakistan

**Keywords:** Ineffective ventilation, H-Type Tracheoesophageal fistula, Ventilation corrective strategies, Chemical pneumonitis

## Abstract

Tracheoesophageal fistula (TEF) with or without associated esophageal atresia (EA) in the neonate is challenging to diagnose and manage its complications like aspiration, respiratory distress, and other associated anomalies. To stabilize, ventilate and prepare for surgical correction, understanding the H-nature of disease and anticipation of problems and their management will improve survival. We present a newborn with tracheoesophageal fistula without atresia from resource-limited settings and lessons we learned from the case.

## INTRODUCTION

Tracheoesophageal fistula with or without atresia manifest in newborns from hours to days after birth and have several classification systems based on the presence or absence of atresia and its location with fistula.^1^ Congenital isolated TEF (H type) is a rare disorder connecting carina or main bronchi at an oblique angle to the esophagus. Pressure changes between both structures can cause air entry into the esophagus or entry of esophageal content into the trachea.^2^ The clinical features are variable; common presentation being recurrent respiratory infections, aspiration during feeding with cyanosis and abdominal distension,^3^ and disappearance during nasogastric tube feeding symptoms.

H-type TEF accounts for about 5% of all trachea-esophageal malformation, with an estimated incidence of about one per hundred thousand live birth.^4^ The early diagnosis of this defect is challenging^5^ and can be delayed because the presentation is non-specific.^6^ However, if diagnosed early, the approach to airway management and invasive ventilation before surgical correction is quite challenging, particularly with the significant defect. We report a case of H-type TEF in a preterm baby complicated by pneumonitis and respiratory failure secondary to ineffective ventilation.

## CASE REPORT

A baby girl weighing 1400grams was born at 33+4weeks of gestation by emergency cesarean section due to fetal distress. An anomaly scan at 22 and 33 weeks was suspicion of esophageal atresia with absent stomach bubble, polyhydramnios with AFI of 32.6cm, and high resistance diastolic flow.

The baby was born in good condition and didn’t require any resuscitation at birth. However, soon after delivery, she developed respiratory distress with excessive salivation requiring frequent oral and nasal suctions. In NICU, oxygen was started via a high-flow nasal cannula, an orogastric tube inserted, which ruled out EA. Its position was confirmed by chest x-ray, infant was kept nil by mouth, parenteral nutrition, and first-line antibiotics for early-onset sepsis initiated.

Preliminary investigations revealed tiny mid-muscular ventricular septal defect (VSD), small Secundum atrial septal defect (ASD), left-sided aortic arch, and small patent ductus arteriosus (PDA). Ultrasound abdomen and blood investigations were unremarkable.

**Fig.1:**
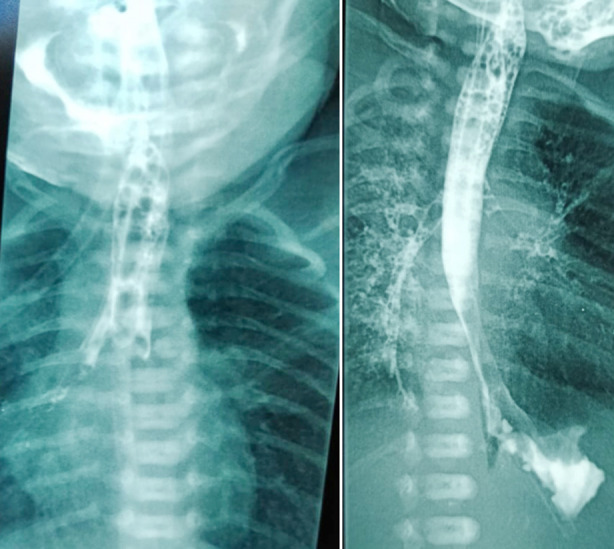
Contrast study showing H-type fistula with spillage of contrast in lungs.

**Fig.2:**
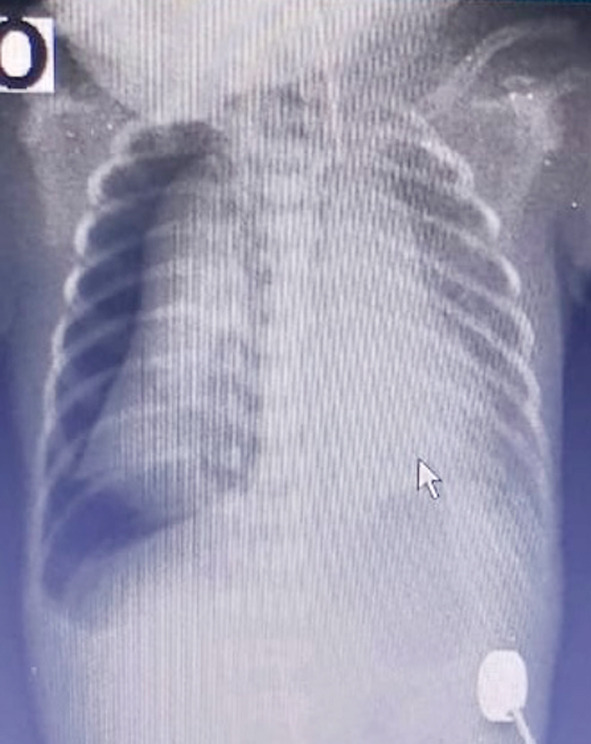
Chest X-rays showing right-sided pneumothorax.

At 24hours of life, fluoroscopy using non-ionic water-soluble contrast confirmed large H-type TEF just above the carina with spillage of contrast into the lungs. The procedure was abandoned immediately due to worsening respiratory distress with persistent desaturation and severe respiratory acidosis requiring intubation and mechanical ventilation. The serial X-rays confirmed bilateral chemical pneumonitis, Fig.1 shows contrast spillage into lungs while Fig.2, shows right sided tension pneumothorax, which was successfully drained. Despite confirming proper placement of endotracheal tube clinically and on serial X-rays, ventilation and oxygenation were unsuccessful due to a large leak of air from fistula to the esophagus with resultant ineffective peak pressure measurement on a ventilator. A nasogastric tube (NGT) was aspirated continuously to decompress the stomach. Micro cuffed endotracheal tubes (ETT) and expertise to use high-frequency ventilator were not available in NICU. However, a brief period of successful ventilation was achieved with the help of foley’s catheter in the esophagus with its balloon inflated in small increments at a frequent distance, till at 9cm when effective ventilation and adequate peak pressures were noted on the ventilator screen (***Video Link:*** https://youtu.be/7KCzB-WE4Ik) without causing excessive distension of the stomach. Though causing a slight tamponade effect at the fistula site, this maneuver was not adequate for ventilation and oxygenation. The baby passed away after a prolonged but unsuccessful resuscitation by approximately 54 hours of age due to Type-2 respiratory failure.

## DISCUSSION

Proper diagnosis and management of TEF in the resource-limited setting are challenging. Airway management and ventilatory strategies are the most critical aspects for TEF management, the goal of which should be to avoid insufflation of gas via fistula into the stomach, causing ineffective ventilation and abdominal distension, thereby pushing the diaphragm up, adding further to impair ventilation, aggravating hypoxemia.^7^ We share this case to highlight the two most important aspects of management, i.e., diagnosis and mechanical ventilation.

### Diagnosis

The use of fiber-optic bronchoscopes to confirm and locate the fistula is critical to avoid complications. Contrast-enhanced studies have the risk of aspiration pneumonia and pulmonary injury are not helpful in delineating the size and precise location.^7^ If performed, all neonatal emergency resuscitation equipment must be ready if needed.^8^ Since fiber-optic bronchoscopes are not available in our setting, one might need to delay diagnostic procedures if the baby is clinically stable to let the baby become more mature. The focus must be directed towards the nutritional intake at this stage.

### Mechanical ventilation

A neonate in respiratory distress should be managed with non-invasive ventilation, although ideally, it is better not to ventilate preoperatively.^9^ Since our patient was suspected of having a large fistula near the carina and low compliance (due to pneumonitis), conventional ventilation with uncuffed ETT failed to prevent excessive air leaks into the stomach and, consequently, ineffective ventilation. The fistula’s occlusion with foleys catheter, although temporally caused tamponade effect, was not secure enough for adequate ventilation. Cuffed ETT and Fogarty catheters have been tried with success in such situations.^8^ However, these are not successful in low carinal fistulae. We advise against attempting such measure early in management to acquire time for stabilization, and emergency ligation of fistula can be performed with definitive repair a week later.

## CONCLUSION

Preoperative bronchoscopy is the investigation of choice which should be done before surgical correction of the fistula with all necessary equipment for resuscitation and ventilation. The major challenge is airway management before ligation of the fistula, especially with large peri-carinal fistulae, due to ineffective ventilation and distension of the stomach, subsequently worsening hypoxemia by pushing the diaphragm. Use of cuffed ETT or catheter (ureteral or Fogarty) can help prevent leak and help achieve adequate ventilation.

### Authors’ Contribution:

**FA, VK, ND** conceived, designed, Literature search & is responsible for the integrity of research.

**ND, VK,** prepared drafting drafts and review after final approval.

**SN, FA** acquisition of data and manuscript writing.

**FA, VK, SN, ND** did manuscript writing.
